# Laser therapy in bone repair in rats: analysis of bone optical density

**DOI:** 10.1590/1413-78522014220200438

**Published:** 2014

**Authors:** Danillo Barbosa, Antonio Guillermo Jose Balbin Villaverde, Emilia Ângela LoschiavoArisawa, Renato Aparecido de Souza

**Affiliations:** 1 Universidade Camilo Castelo Branco, São José dos Campos, SP, Brasil, Universidade Camilo Castelo Branco, São José dos Campos, SP, Brasil; 2 Universidade do Vale do Paraíba, São José dos Campos, SP, Brasil, Universidade do Vale do Paraíba, São José dos Campos, SP, Brasil; 3 Instituto Federal de Educação, Ciência e Tecnologia, Muzambinho, MG, Brasil, Instituto Federal de Educação, Ciência e Tecnologia, Muzambinho, MG, Brasil

**Keywords:** Fractures, bone, Osteotomy, Technology, radiologic, Fracture healing, Rats, wistar

## Abstract

**OBJECTIVE:**

To investigate, by digital radiology, the bone regeneration process in rats submitted to femoral osteotomy and treated with low power laser therapy.

**METHODS:**

Forty-five Wistar rats were subjected to transverse osteotomy of the right femur and divided randomly into three experimental groups (n = 15): animals not treated with laser therapy G (C), animals that received laser therapy with λ: 660nm G (660nm) and animals that received laser therapy with λ: 830nm G (830nm). Animals were sacrificed after 7, 14 and 21 days. The bone calluses were evaluated by digital X-ray at 65 kVp, 7mA and 0.032 s exposures.

**RESULTS:**

The values obtained were submitted to variance analysis (ANOVA) followed by the Tukey-Kramer test. The significance level adopted was 5%. The groups G (C), G (660nm), and G (830nm) at the 7^th^ day showed a significant bone development, with p <0.0116; the groups G (C), G (660nm), and G (830nm) at the 14^th^ day showed values of p <0.0001; at the 21^st^ day,a higher degree of bone repair were observed in group G (830nm), and G (660nm), with p <0.0169.

**CONCLUSION:**

Based on the radiographic findings, G (830nm) showed more complete bone regeneration, as shown in the gray shades of the images. ***Level of Evidence II, Individual Study With Experimental Design.***

## INTRODUCTION

The need of restoration and proper bone healing often hinders the work of health professionals involved in the rehabilitation process of patients with bone fractures.^1^ In order to reduce the significant disability associated with this bone disorder, as well as its high socioeconomic cost, a variety of therapeutic interventions have been shown to stimulate bone repair, such as the use of low power laser therapy (LPLT).^2^ ^-^ ^3^

LPLT hits non-thermal reactions of light with the tissue, causing photochemical effects, i.e. low radiation power density (PD) of 0.01 to 1W/cm2 and also low energy density (ED) of 1 to 10 J/cm^2^ are applied to the biological tissues producing a small non-significant increase in tissue temperature.^4^ ^-^ ^5^ This type of therapy has been investigated as to its effects associated with cell proliferation and repair of various biological tissues, including bone tissue. Some authors reported that LPLT could accelerate bone formation by increasing osteoblastic activity,^6^ ^-^ ^7^ vascularization,^8^ ^-^ ^9^ organization of collagen fibers,^10^ and intracellular ATP levels.^11^

Although it has been demonstrated the ability of LPLT to promote osteogenesis in vivo and in vitro and to reduce the time of fracture consolidation by biochemical stimulatory effects, it has been attributed to LPLT on bone tissue are still controversal.^12^ In a review study,^12^ it has been attributed to conflicting results regarding LPLT and bone healing the therapeutic LPLT parameters described in the experimental trials. It is likely that bone regeneration does not depend solely on the total dose of radiation, but also the time, mode of irradiation, and wavelength of the laser beam. In the latter case, it was hypothesized that the photochemical and photophysical properties of some wavelengths are primarily responsible for tissue response. While the laser radiation in the visible spectrum has mitochondrial pathway activation, the laser radiation in the infrared spectrum (invisible) has an action on the chromophores of the cellular membrane.^13^

Typically, studies looking at the influence of LPLT on bone healing used the following methodologies of analysis: optical microscopy,^14^ morphometry,^15^ imunohistochemistry,^16^ and biomechanical tests.^17^ However, radiographic examination is an important clinical tool for bone analysis because it is noninvasive, is low cost and fast in collecting and interpreting results. A radiographic method was investigated for measuring bone density based on scanned grayscale radiographic image (bone optical density), and concluded that this method shows similar results to the histological findings, regarding the characterization of the mineral component.^18^ Through this methodology^19^ the effect of LPLT (λ: 830nm) on tibia fractures in rabbits was evaluated and showed an increase in callus bone volume and bone density associated to the therapy. 

Considering the enormous biotechnological advances in healthcare, LPLT is presented today as a popular and easily available tool to attempt to obtain adequate bone repair. However, in order to give more support and more effective therapeutic outcomes, more research is needed to establish and standardize best irradiation parameters, as well as new analytical tools. In this context, the aim of this study was to evaluate by optical densitometry the bone regeneration process in rats with femoral osteotomy treated with LPLT (λ: 660 nm and λ: 830 nm) during 7, 14, and 21 days.

## MATERIALS AND METHODS

In this study the standards for educational and scientific practice of vivisection of animals ( Law 6638 from May 8, 1979 ) were adopted, and the procedure was approved by the Ethics and Research Committee of Universidade do Vale do Paraíba under protocol n° L213 -2005. Forty five young adult male Wistar rats (*Rathus Norvegicus*) weighing between 250g and 270g , kept in an environment with controlled light (12 hours cycles light and dark) and controlled temperature (23 º C), with animal food and water *ad libitum* were used. The study was developed in the vivarium of Laboratory of Physiology and Pharmacodynamics, Instituto de Pesquisa e Desenvolvimento da Universidade do Vale do Paraíba, São José dos Campos, SP, Brazil (Univap). The animals were randomly divided into three groups (n = 15) and subdivided into three groups (n = 5), according to experimental intervention time. All animals underwent surgical procedure for inducing bone defect (osteotomy) and were sacrificed at 7, 14, and 21 days after surgery. In group I (control group), the bone defect was induced, however, the animals received no treatment; animals of group II were treated with LPLT in the red region (λ: 660nm); and animals in group III received LPLT in the infrared spectrum (λ: 830 nm). (Table 1)

**Table 1:** Description of groups, sample (n), procedure and treatment. Table 1. Description of groups, sample (n), procedure and treatment.

Group	n	Procedure	Experimental Times (days)			Treatment
			7	14	21	
I (control)	15	Femoral Osteotomy	5	5	5	None
II (λ: 660nm)	15	Femoral Osteotomy	5	5	5	48h - 48h
III (λ: 830nm)	15	Femoral Osteotomy	5	5	5	48h - 48h

### Surgical Procedure

Animals were previously medicated with an intramuscular injection of acepromazine (0.02 ml/kg) and butorphanol (0.01 ml/kg). After 15 minutes the anesthetic Zoletil^(r)^ (1.0 ml/kg) was administered via intramuscular injection in the medial region of the quadriceps. After anesthesia, animals were shaved in the right femoral region with a razor and a topical iodine solution was applied. As a biosecurity procedure, a sterile TNT (nonwoven fabric) in the surgery field region was used. With a scalpel blade, an incision of approximately 3 cm length in the surface of the skin and muscle incision was performed and separated soft tissues and periosteum, exposing the right femoral region. The osteotomy was performed with 2.8 mm diameter trephine drill, with the aid of low-speed electric motor (Dentec^(r)^ 405N) at a speed of 1100 rpm and frequency of 0.25 s, at constant and copious irrigation with saline throughout the surgical procedure. After the osteotomy at the right femur, the muscle layers were sutured with resorbable wire and the skin with sterile silk thread.

**Figure 1:**
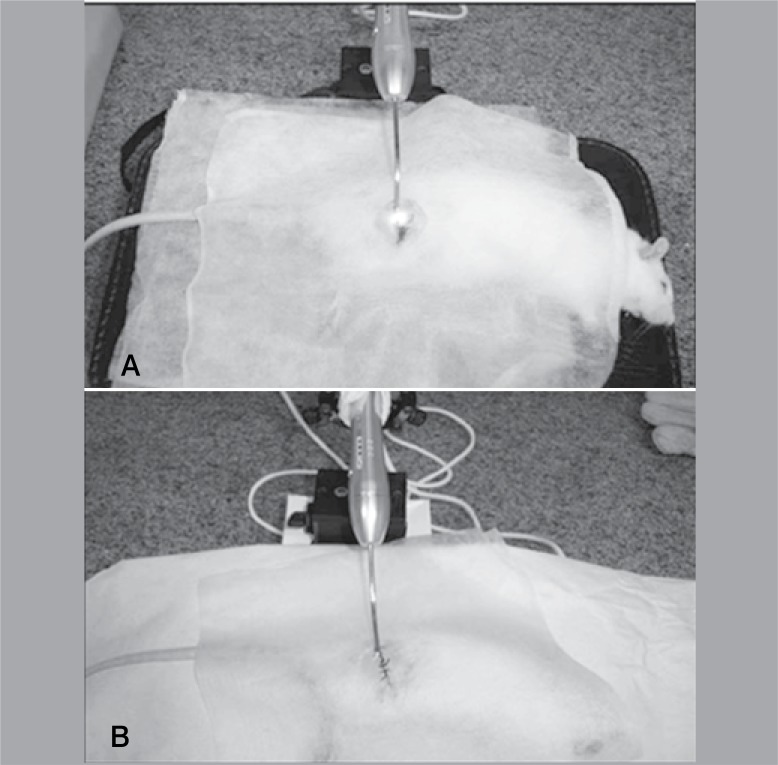
First laser application immediately after surgery (osteotomy). (A) Animal from Group II receiving ? 660 nm LPLT (see bright spot). (B) Animal from Group III receiving ? 830nm LPLT (invisible wavelength).

LPLT was performed in groups II and III immediately after osteotomy and every 48 hours for 7, 14, and 21 days. (Figure 1) A laser device Flash Lase III (DMC Equipment Ltda, São Carlos, SP, Brazil), which operates with laser light at a wavelength between 660-690 nm was used (red laser; Active Mode: InGaAIP) and between 790-830 nm (infrared laser; Active Mode: AsGaAl). The laser was applied in contact with the operated skin region perpendicularly at only one point, according to the irradiation protocol described in Table 2.

**Table 2:** Lasers irradiation protocols.

Irradiation Parameters	Laser 660 nm	Laser 830 nm
Energy Density	4 J/cm^2^	4 J/cm^2^
Power	100mW	100mW
Time	40 s	40 s
Irradiated area	1 cm^2^	1 cm^2^

### Bone Optics Densitometry

For radiographic images of femurs the X-ray machine digital765 DC Gendex^(r)^ was used with the following parameters: 65 kVp, 7mA, and 0,032 s exposure time. For capturing images, a direct digital radiography system which employs the charge-coupled device (CCD) RVG (Trophy Radiologie, Vincennes, Toulose, France) was used. The CCD sensor was attached to a table with the X-ray cylinder device positioned at a focal distance of 40 cm, so that the central X-ray beam focus perpendicularly to the sensor. Each anatomical specimen was placed on the sensor with the bone defect occupying the central portion. The images were stored in TIFF format in a standard resolution. Thereafter, the optical density analysis was performed in an Image Tool 2:03^(r)^ software, using the Histogram tool (optical density versus the number of pixels), demarcating the central region of the bone defect. Thus, we obtained a two-dimensional graph, providing the grayscale values ​​of the radiographic image. Two readings of mean density of each radiographic image were performed by the same examiner, at an interval of one week each.

### Statistical analysis

Results are expressed as mean plus or minus standard deviation. Analysis of variance (ANOVA) among experimental groups in the different experimental periods was used. The Tukey *post hoc* test for multiple comparisons was used to identify specific differences in the variables that met the criterion for pre-established statistical significance (p <0.05).

## RESULTS

**Figure 2:**
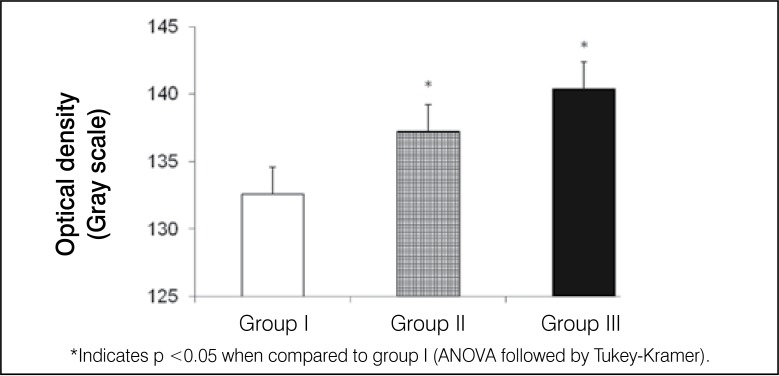
Mean optical density of the osteotomized femur after 7 days of treatment with low-power laser therapy (LPLT). Group I: Control (n = 5); Group II: animals treated with LPLT (?: 660nm) (n = 5); Group III: animals treated with LPLT (?: 830nm) (n = 5).

After 7 days of experimental intervention a statistically significant increase (p <0.05) in the average optical density (grayscale) in groups II and III was observed when compared to group I (3.4% and 5.8 %, respectively). (Figure 2) No differences were found between the groups treated with LPLT (II and III) at the end of the experimental period (p> 0.05).

**Figure 3:**
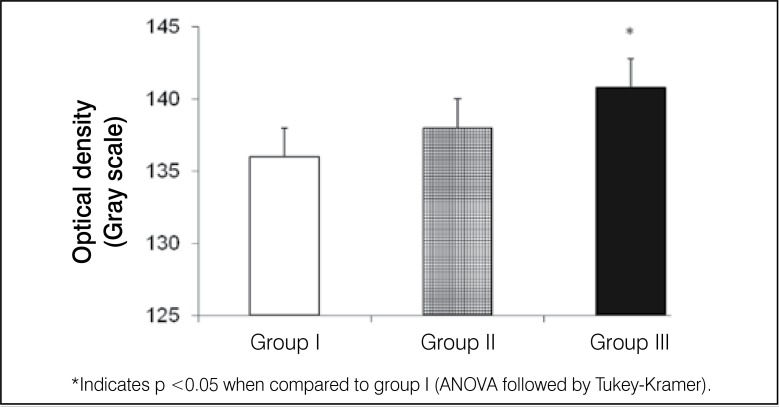
Mean optical density of the osteotomized femur after 14 days of treatment with low-power laser therapy (LPLT). Group I: Control (n = 5); Group II: animals treated with LPLT (?: 660nm) (n = 5); Group III: animals treated with LPLT (?: 830nm) (n = 5).

At the end of 14 days of treatment with LPLT it was observed that only the average optical density of animals in group III was statistically different (p <0.05) than the value found for group I (3% higher). (Figure 3) In that trial period no differences were found between the groups treated with LPLT (II and III) (p> 0.05) and also between the mean optical density values of group I and II (138 ± 2.4 *versus *136 ± 1.8).

**Figure 4:**
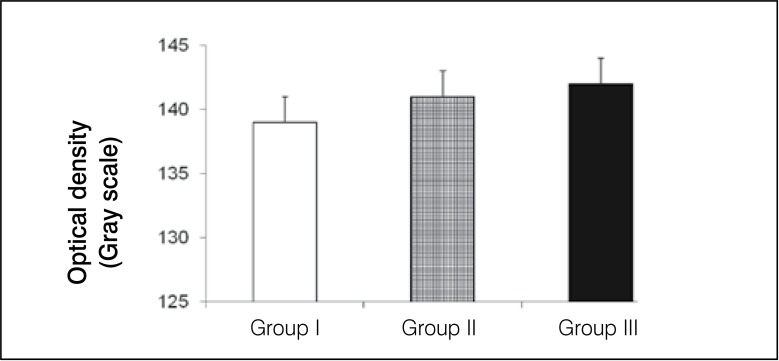
Mean optical density of the osteotomized femur after 21 days of treatment with low-power laser therapy (LPLT). Group I: Control (n = 5); Group II: animals treated with LPLT (?: 660nm) (n = 5); Group III: animals treated with LPLT (?: 830nm) (n = 5).

By analyzing the average optical density of the osteotomized femurs after 21 days, no significant differences were found between the experimental groups (p> 0.05). (Figure 4) While the group I (control) showed a mean of 139 ± 2.3, the groups treated with LPLT, showed average values of 141 ± 2.5 (group II) and 142 ± 2.1 (group III).

## DISCUSSION

It was observed that the bone defects which underwent treatment with laser in the infrared spectrum (Group III) showed higher optical densities compared to the control group (Group I) after 7 and 14 days. (Figures 2 and 3) This fact characterizes increased bone deposition in group III and suggests an accelerated repair associated with LPLT with the 830nm wavelength. This finding is in agreement with the results presented in the literature. The effect of LPLT ( AsGaAl, 830nm,^20^ 40mW, continuous mode, beam diameter ~0.6 mm, 16 J/cm^2^ per session) on bone repair of rat femurs was histologically evaluated. LPLT was started immediately after surgery and repeated every 48 hours for 15, 21, and 30 days. The results of this research demonstrated increased collagen in the early stages of bone repair (15 days) and increased well organized trabecular bone after 30 days in the irradiated animals. Therefore, it was concluded that LPLT in the infrared region (λ: 830 nm) caused a positive effect on biomodulation of bone remodeling. In another study, LPLT has been used with 830 nm wavelength laser (AsGaAI) aiming to investigate the osseointegration ability of this therapy that associated with a titanium alloy. LPLT was applied immediately after surgery and continued for 10 consecutive days. After eight weeks mechanical, morphologic and radiographic tests revealed that LPLT has generated positive effects on the the bone repair process.^21^ A study that evaluated the effects of LPLT on human osteoblastic cells used daily doses of AsGaAI laser irradiation (830nm, 90mW, energy densities from 1.7 to 25.1 J/cm^2)^ for 10 days, and increased intracellular calcium indicated that these cells responded appropriately to LPLT.^22^

A higher optical bone density in animals treated for 7 days with LPLT, regardless the wavelength used demonstrates the effect of biomodulator effect of this therapy in the early stages of bone repair. During this phase, the effectiveness of laser action appears to be related to its ability to (a) promote proliferation of endothelial cells which leads to the formation of a new rich vascular network, ensuring blood supply to the repair process.^8^ ^-^ ^9^ (b) stimulate fibroblast favoring collagen synthesis and bone growth,^10^ (c) stimulate osteoprogenitor cells, osteoblasts and osteoclasts, contributing to bone remodeling^6^ ^-^ ^7^ and (d) increased synthesis of DNA and RNA, indicating an effect on cell growth and protein synthesis.^11^

The LPLT with visible radiation (Group II) promoted only greater optical density compared to the control group (Group I) in the initial repair phase (7 treatment days). It has been described in the literature that red spectrum wavelength LPLT, provided that it is administered in the early stages of bone inflammatory response is able to accelerate the repair processes. He-Ne laser (6 mW at a dose of 31 J/cm^2^, for about 3 minutes) was used,^11^ in bone defects produced in tibias, on the 5^th^ and 6^th^ day postoperatively. The authors observed that LPLT caused an increase in the number of osteoblasts, increased levels of alkaline phosphatase, and faster bone healing than animals that did not receive low-power laser treatment. The effect of He-Ne laser (35 mW ) on bone healing of fractures of the rat tibia was assessed^11^. The laser was applied for 30 minutes daily during 14 days. The results obtained through biomechanical methods showed that laser improved bone regeneration. The authors concluded that the difference in results between groups may be due to the characteristics of the bone callus. The control group presented a fibrocartilaginous callus, whereas the laser group showed a more mature and resistant callus. In another study^7^ it has been shown that the He-Ne laser promoted proliferation and maturation of human osteoblasts *in vitro* with increased levels of the bone enzymes alkaline phosphatase, osteopontin and sialoprotein.

However, other studies using LPLT with visible radiation was unsuccessful in bone repair, since no significant change in the evolution of bone repair was found when the low power He- Ne laser was used in tibia fractures.^8^ Similarly, authors warned that bone regeneration depends on the applied dose and the time interval between applications of laser.^9^ In this regard, the authors^8^ ^,^ ^9^ had positive results when the He- Ne laser was applied early in the first days after surgery, in at least 3 doses. In the present study it was hypothesized that the efficacy of bone regeneration after 7 days in group II would be related to the earliness of the first application, which occurred immediately after inducing bone defect.

## CONCLUSION

Based on the results obtained and according to the methodology employed in this study, it is concluded that LPLT was able to promote bone regeneration after 7 days of treatment regardless of the two wavelength used (660nm or 830nm). However, after 14 days of treatment bone regeneration is more efficient with infrared wavelength (830nm) laser. Furthermore, optical bone density measurement appears to be an appropriate technique to observe the evolution of bone repair.
